# Supplementary material for: Limitation by a shared mutualist promotes coexistence of multiple competing partners
Source: Nat Commun. 2021 Jan 27;12:619. doi: 10.1038/s41467-021-20922-0 (PMC7840915; doi:10.1038/s41467-021-20922-0)
Supplement: Supplementary file 1 — Supplementary Information [file 41467_2021_20922_MOESM1_ESM.pdf]

Supplementary Information for Hammarlund et al., *Limitation by a shared mutualist promotes coexistence of multiple competing partners*

Contents

Supplementary Figure 1: Amino acid requirements of Em and Er

Supplementary Figure 2: Monoculture final yields

Supplementary Figure 3: Coculture final yields

Supplementary Figure 4: Er competitively excludes Em regardless of whether Smr is present

Supplementary Figure 5: In a lactose environment, Smr stably coexists with Em and Er

Supplementary Figure 6: Competitive exclusion and coexistence over multiple transfers

Supplementary Figure 7: Initial and final densities of Em Er Smr communities in lactose media across a range of initial Em frequencies

Supplementary Table 1: Model parameters and default values

Supplementary Figure 8: Coexistence is robust to changes in initial Smr abundance

Supplementary Figure 9: Dynamics of strains and all resources when Em starts rare

Supplementary Figure 10: Dynamics of strains and all resources when Er starts rare

Supplementary Figure 11: Coexistence is robust to changes in amino acid depletion rates

Supplementary Figure 12: Coexistence is robust to depletion of both amino acids by both *E. coli* strains

Supplementary Figure 13: If *S. enterica* provides methionine to two different methionine-requiring *E. coli* strains, there is no coexistence, even if Sm grows more slowly

Supplementary Table 2: Model parameters and default values for three *E. coli* strain community

Supplementary Figure 14: In the three *E. coli* strain community, the rare *E. coli* strain's amino acid is abundant.

Supplementary Note 1: Coexistence in a Chemostat

Supplementary Figure 1 | Amino acid requirements of Em and Er. Final yields (OD600) of **a**, Em across a range of methionine concentrations and **b**, Er monocultures across a range of arginine concentrations, in lactose minimal media. Cultures were inoculated with log-phase culture and grown until stationary phase was reached, then OD was measured (see Methods for growth conditions and OD measurements). Yields plateau at 100 μM of methionine for Em and between 100 μM and 500 μM of arginine for Er, suggesting that Em requires slightly less methionine than Er requires arginine to reach its maximum yield. We chose 250μM as the concentration of both amino acids for our lactose minimal media supplemented with arginine and methionine because yields saturate around this concentration, indicating that lactose sets carrying capacity rather than the amino acid.

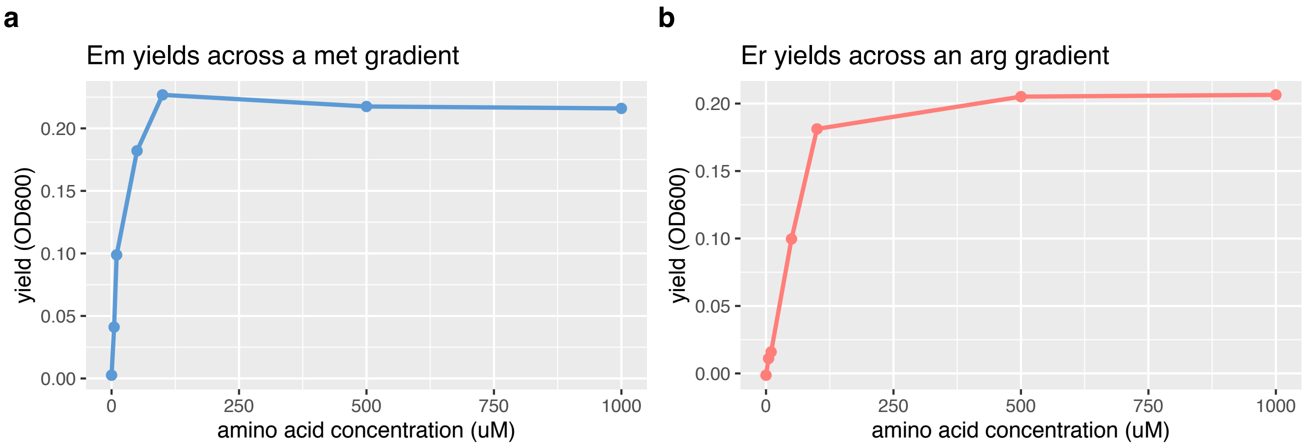

Supplementary Figure 2 | Monoculture final yields. Yields (CFU/ml) of Em, Er, and Smr in monoculture, measured by diluting and plating on selective media. Em and Er were grown in lactose media with methionine and arginine, and Smr in acetate media (see Methods).

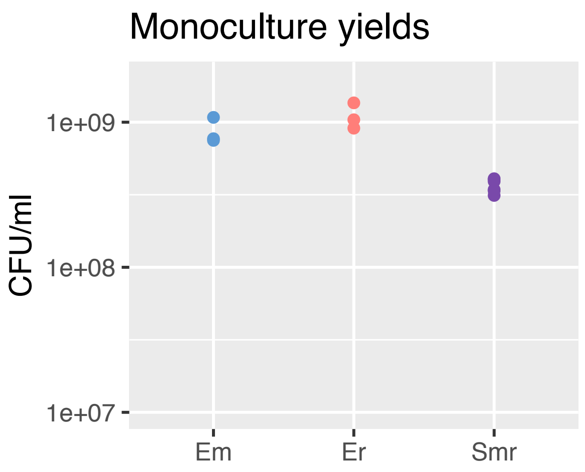

Supplementary Figure 3 | Coculture final yields. a, Yields (CFU/ml) of Em and Smr from Em+Smr cocultures, grow in lactose minimal media, and measured by diluting and plating on selective media. **b**, Yields (CFU/ml) of Er and Smr from Er+Smr cocultures, also grown in lactose media and diluted and plated to count colonies.

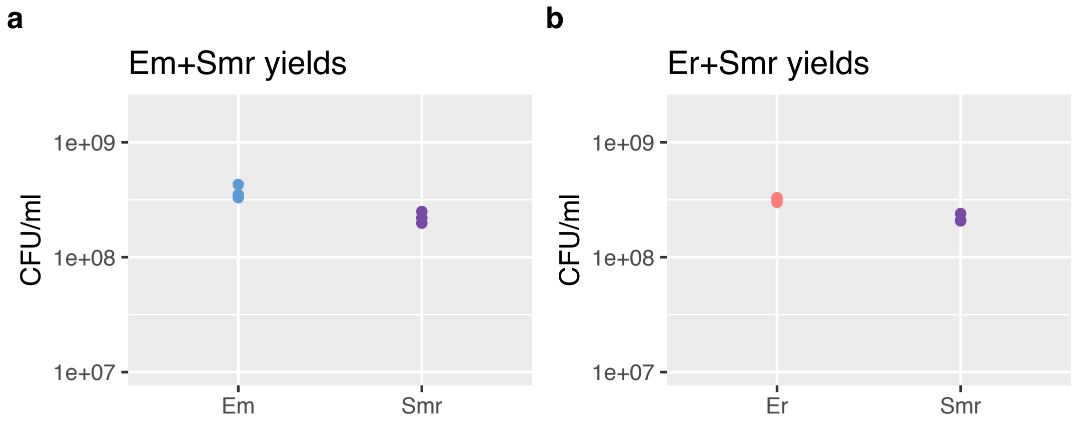

Supplementary Figure 4 | Er competitively excludes Em regardless of whether Smr is present. Em and Er cocultures were grown in lactose + methionine + arginine media with and without Smr, with a starting Em frequency of 0.006. When present, Smr initially made up 16% of the total community density. Em decreased from an initial frequency of 0.006 both when Smr is absent (data also shown in Fig. 2d) or present. Triangles indicate that this experiment was performed with batch B of the mutual invasibility experiments.

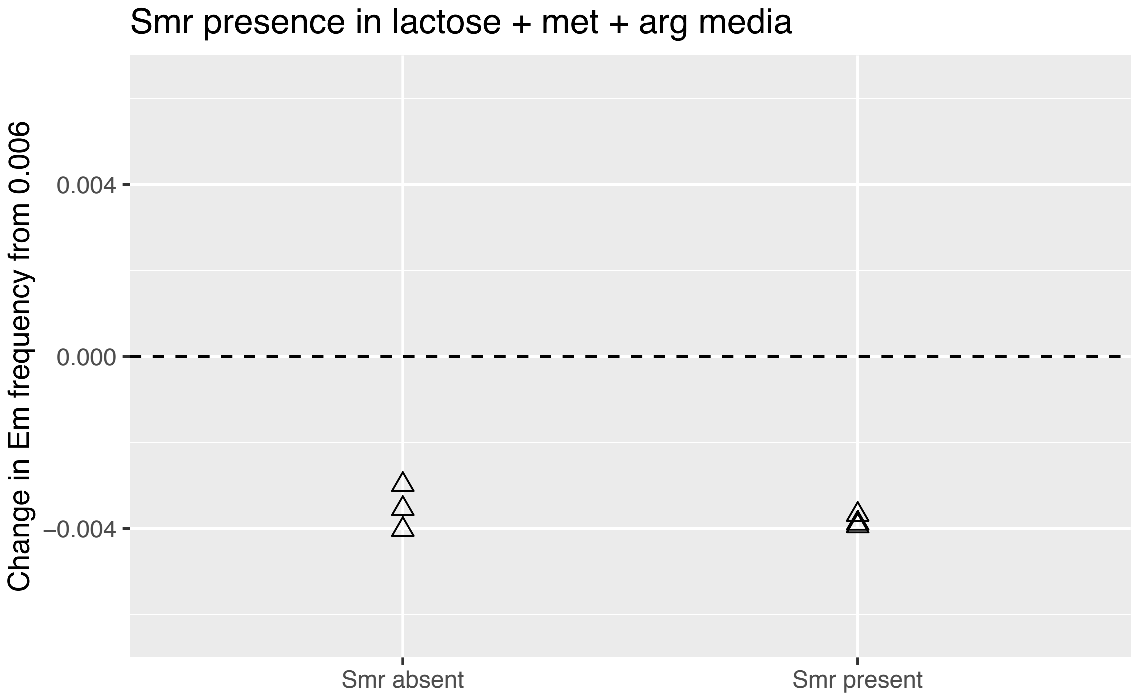

Supplementary Figure 5 | Competitive exclusion and coexistence over multiple transfers. a, In lactose + methionine + arginine media, Er outcompetes Em over multiple growth cycles (Batch C, square points). **b**, In lactose media with Smr, Em is maintained at an intermediate frequency (Batch D, square points). Cultures were diluted 100-fold into fresh media after each two-day growth period, and plated to count colonies. At the timepoint 5 plating, we observed a high frequency of prototrophic colonies derived from the Er strain, suggesting that prototrophic mutants arose and increased to a high frequency during the experiment. To be conservative, we recommend disregarding data after timepoint 2. The evolutionary lability of this system makes mutual invasibility experiments over one growth cycle a better method to assess coexistence.

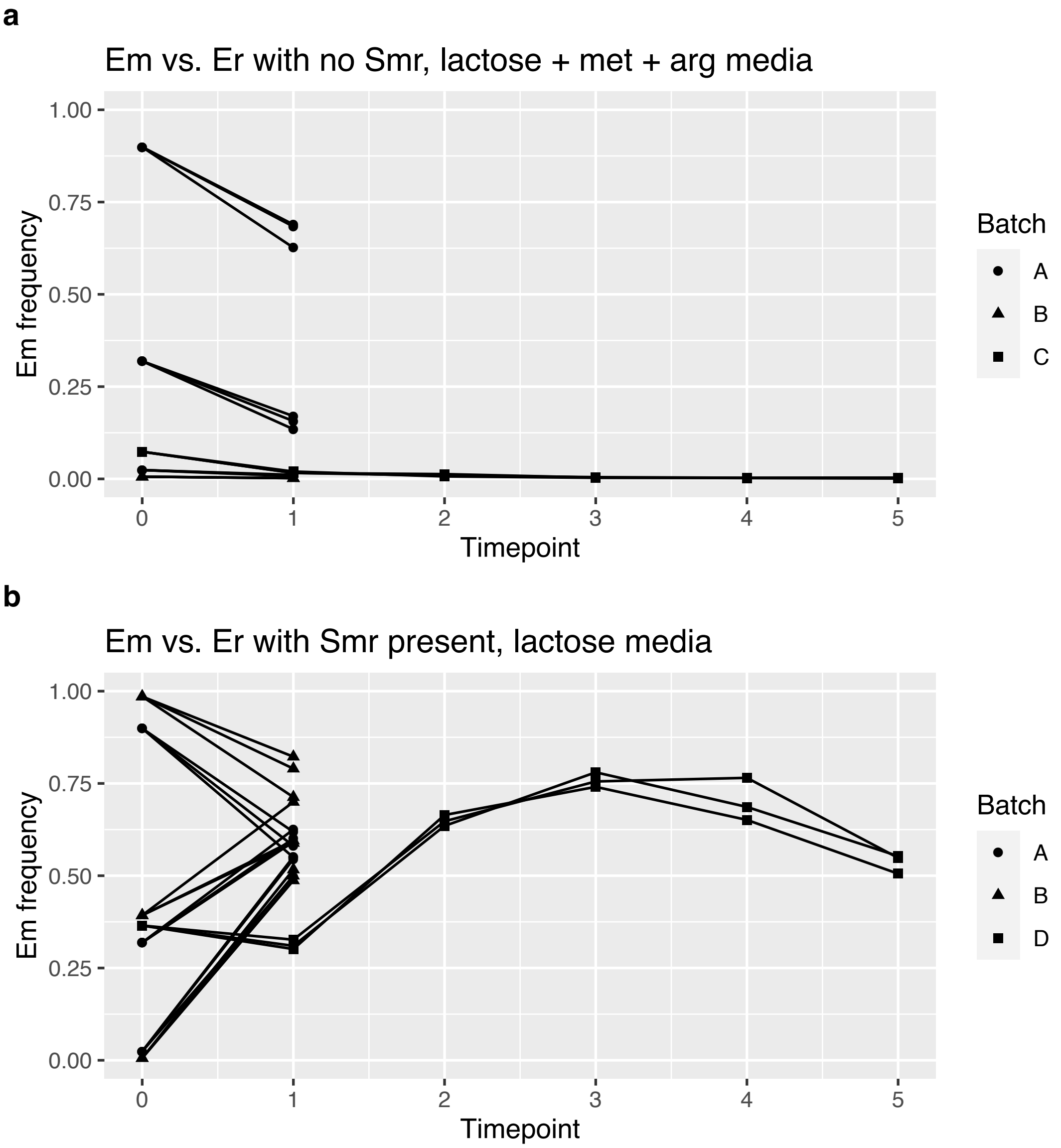

Supplementary Figure 6 | In a lactose environment, Smr stably coexists with Em and Er. Smr frequency (Smr / (Em + Er + Smr)) is shown at the start (Timepoint 0) and end (Timepoint 1) of growth. Smr decreases when initially common and increases when initially rare, indicating that Smr's presence is stable. Averages of three biological replicates from two experimental batches are shown.

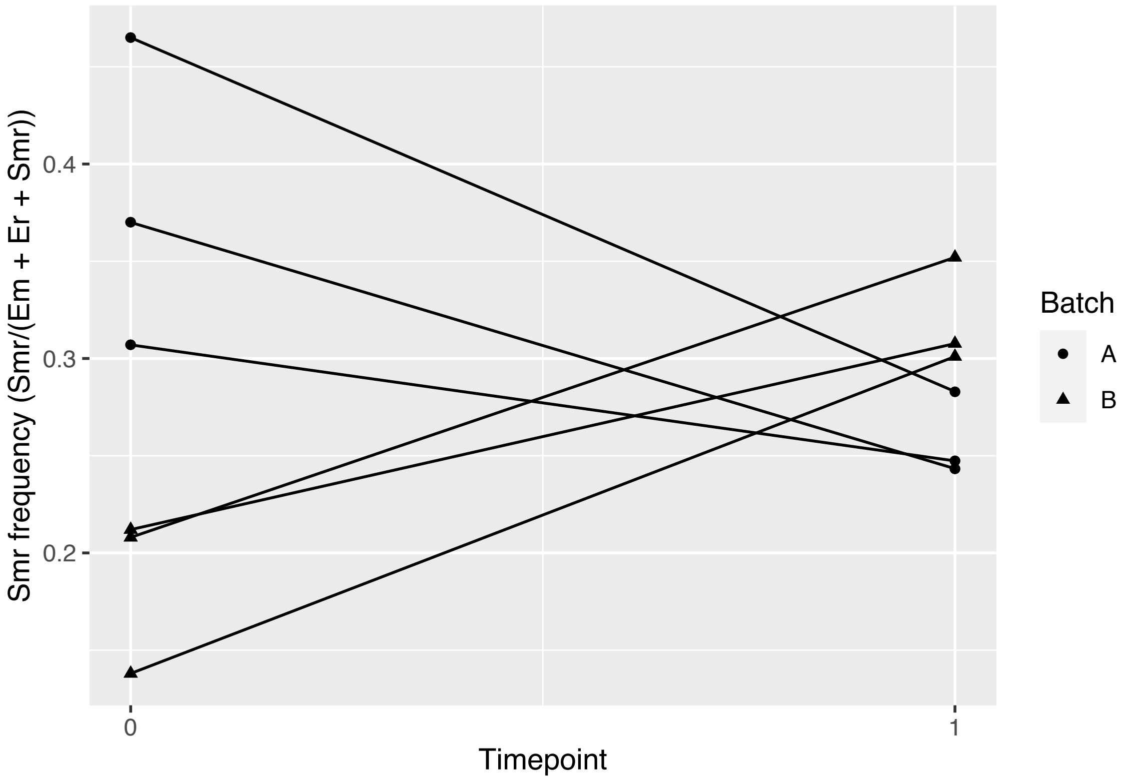

Supplementary Figure 7 | Initial and final densities of Em Er Smr communities in lactose media across a range of initial Em frequencies. Initial (timepoint 0) and final (timepoint 1) CFU/ml of Em, Er, Smr at different Em starting frequencies, shown on the panel titles. Three biological replicates were performed for each strain (points are slightly jittered), and the experiments were performed in two batches, indicated by different shapes. These data correspond to Fig. 2e in the main text.

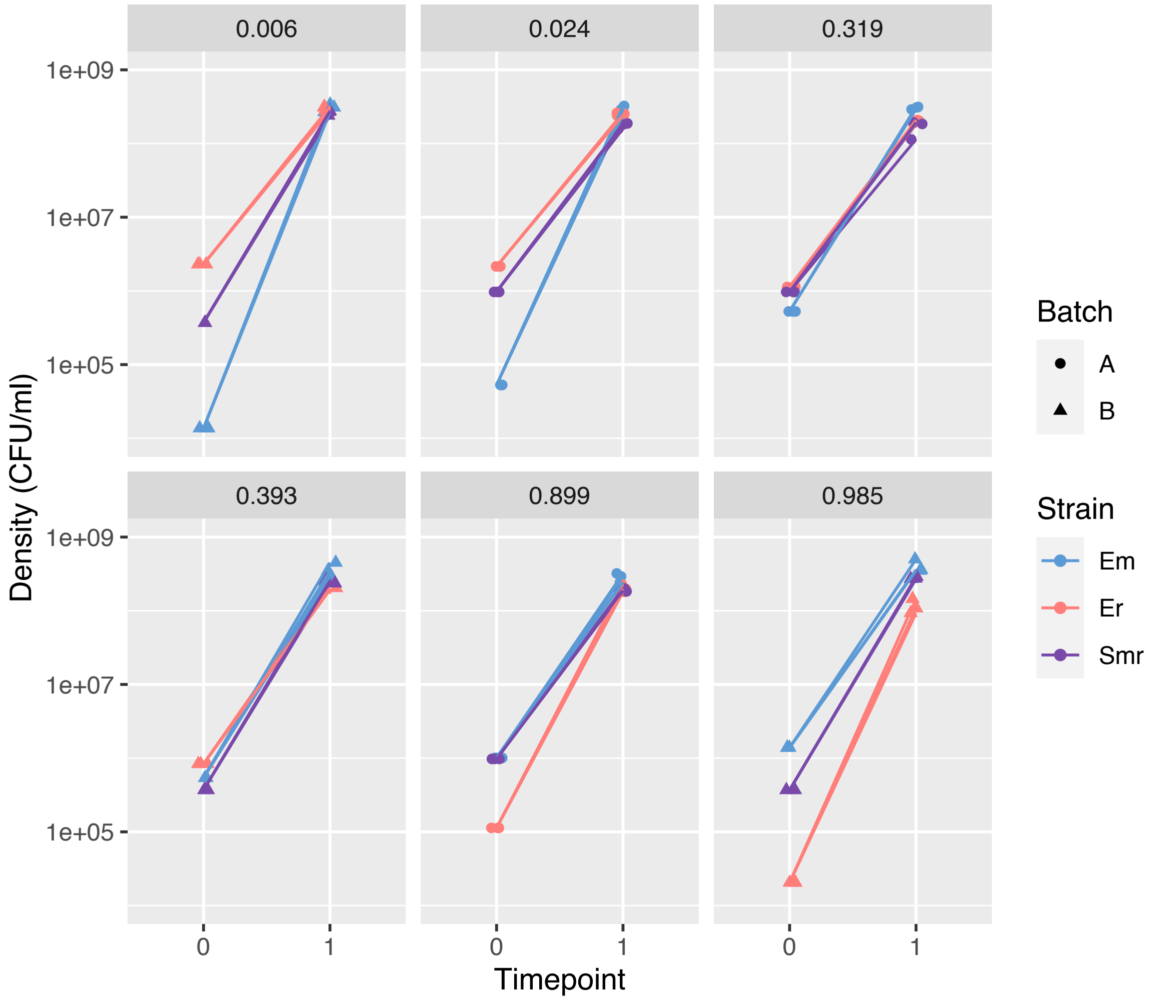

Supplementary Table 1 | Model parameters and default values. Explanation of parameter names, units, defaults, and biological interpretations.

Parameter or state variable	Units	Default or initial value[1]	Name	Biological Interpretation
Smr	Cells/ml	100 at start	Smr cell density	*S. enterica* methionine and arginine producer population size
Em	Cells/ml	variable at start	Em cell density	*E. coli* methionine auxotroph population size
Er	Cells/ml	variable at start	Er cell density	*E. coli* arginine auxotroph population size
lcts	Cells/ml	1000 at start	lactose	The concentration of lactose that can produce a certain cell density once consumed (i.e. cell equivalents)[2]
met	Cells/ml	1 at start	methionine	The concentration of methionine that can produce a certain cell density once consumed (i.e. cell equivalents)[2]
arg	Cells/ml	1 at start	arginine	The concentration of arginine that can produce a certain cell density once consumed (i.e. cell equivalents)[2]
ac	Cells/ml	1 at start	acetate	The concentration of acetate that can produce a certain cell density once consumed (i.e. cell equivalents)[2]
μ_{Smr}	1/Timestep	0.5	Smr growth rate	Maximum growth rate of Smr
μ_{Em}	1/Timestep	1.0	Em growth rate	Maximum growth rate of Em
μ_{Er}	1/Timestep	1.1	Er growth rate	Maximum growth rate of Er
$k_{Em\ lcts}$<br>$k_{Er\ lcts}$<br>$k_{Em\ met}$<br>$k_{Er\ arg}$<br>$k_{Smr\ ac}$	Cells/ml	0.01	Monod half-saturation constants	The value of k determines the resource concentration where growth rate is half-maximum
$p_{Smr\ met}$<br>$p_{Smr\ arg}$<br>$p_{Em\ ac}$<br>$p_{Er\ ac}$	Cells/cell	1.0001	Resource production rates	The amount of the specified resource produced by the specified strain, in cell equivalents per cell

$c_{Em\ met}$ [3]<br>$c_{Er\ arg}$ [3]	Unitless	1	Amino acid depletion rate	A term that scales the depletion of methionine by Em and arginine by Er, relative to a baseline depletion of 1
$c_{Em\ arg}$ [4]<br>$c_{Er\ met}$ [4]	Unitless	0	Depletion rate of the other *E. coli* strain's required amino acid	A term that scales the depletion of arginine by Em and methionine by Er, relative to a baseline depletion of 0

[1] Analyses with altered parameter values are stated in the results and figure legends.

[2] Resources are defined in terms of the amount of cells that can be produced by that concentration ("cell equivalents"). Specifically, resource concentrations (mmol resource/ml) are multiplied by a conversion parameter (1 cell/mmol resource) to result in cells/ml. For example, 1000 cell equivalents of lactose means that *E. coli* would grow to a density of 1000 cells/ml.

[3] Amino acid equations using these depletion rates are shown below with Supplementary Figure 10.

[4] Amino acid equations using these depletion rates are shown below with Supplementary Figure 11.

Supplementary Figure 8 | Coexistence is robust to changes in initial Smr abundance. Em, Er and Smr quickly reach equilibrium densities when grown over multiple growth cycles with a 100-fold dilution at each transfer. Two initial Em frequencies are shown in the two different rows, and three different initial Smr frequencies are shown in columns. Both Em and Er increase in frequency when started rare (compare rows), and coexistence is not affected by the initial Smr abundance (compare columns).

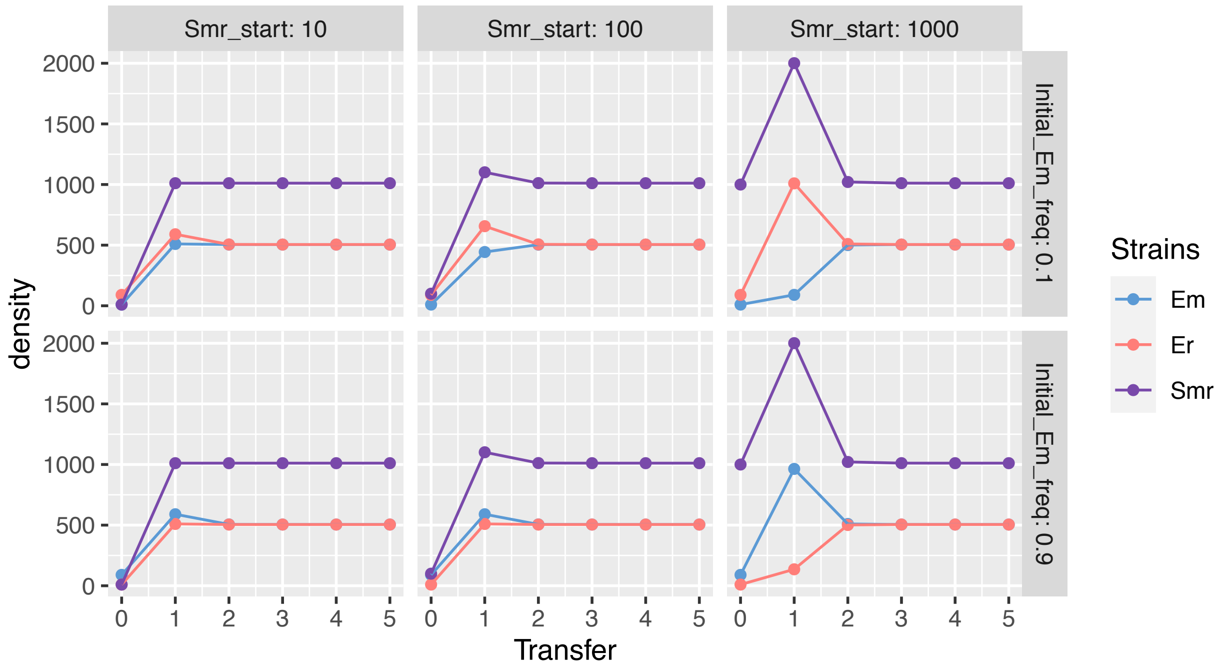

Supplementary Figure 9 | Dynamics of strains and all resources when Em starts rare.
Growth curves and resource plots at the point labeled "**c**" in Fig. 3b.

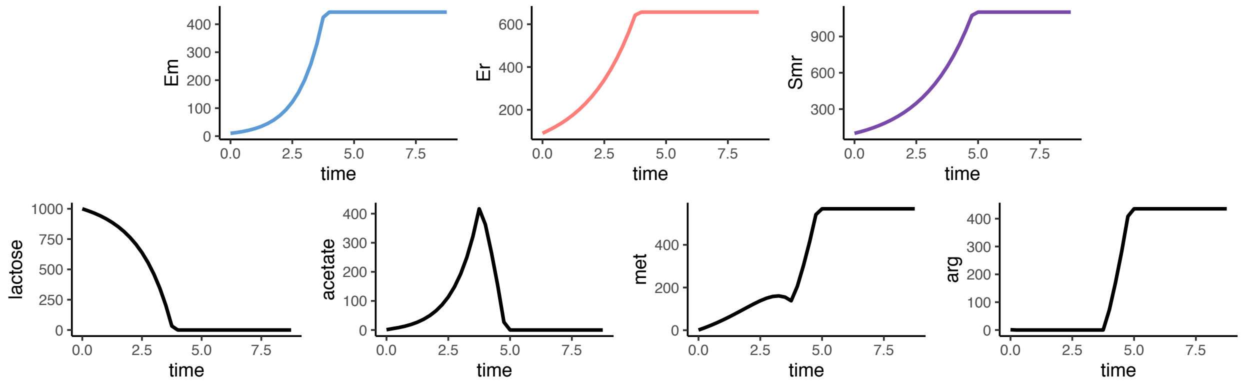

Supplementary Figure 10 | Dynamics of strains and all resources when Er starts rare. Growth curves and resource plots at the point labeled "**d**" in Fig. 3b.

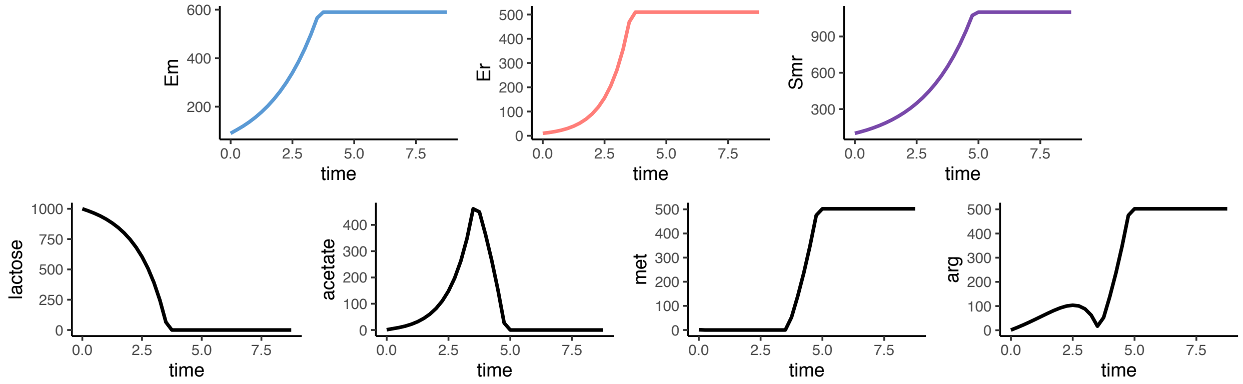

Supplementary Figure 11 | Coexistence is robust to changes in amino acid depletion rates. Depletion rates $c_{\text{Em met}}$ and $c_{\text{Er arg}}$ adjust the rate at which the *E. coli* strains deplete their required amino acids. The default value is 1. A change in the depletion rate does not directly affect the *E. coli* strains' growth (i.e. the equations for Em and Er are unchanged). See Supplementary Table 1 for further description of these parameters and the equations below for how they are incorporated into the model. For all simulations, Em begins at a frequency of 0.1 **a**, Effects of altering the met depletion rate by Em only. Em is able to invade from rare (10%) below a depletion rate of 9. **b**, Effects of altering the arg depletion rate by Er only. Coexistence is maintained except at values of $c_{\text{Er arg}}$ below 0.26. **c**, The effects of altering both depletion rates, keeping them equal. Coexistence is possible above $c_{\text{Em met}} = c_{\text{Er arg}} = 0.26$. For all plots, the dotted vertical line at x = 1 shows the default value of the depletion rate.

Altered equations for amino acids using these depletion rates (in bold):

$$\frac{d\,met}{dt} = -\left(\frac{dEm}{dt} \times \boldsymbol{c_{Em\,met}}\right) + \left(p_{Smr\,met} \times \frac{dSmr}{dt}\right)$$

$$\frac{d\,arg}{dt} = -\left(\frac{dEr}{dt} \times \boldsymbol{c_{Er\,arg}}\right) + \left(p_{Smr\,arg} \times \frac{dSmr}{dt}\right)$$

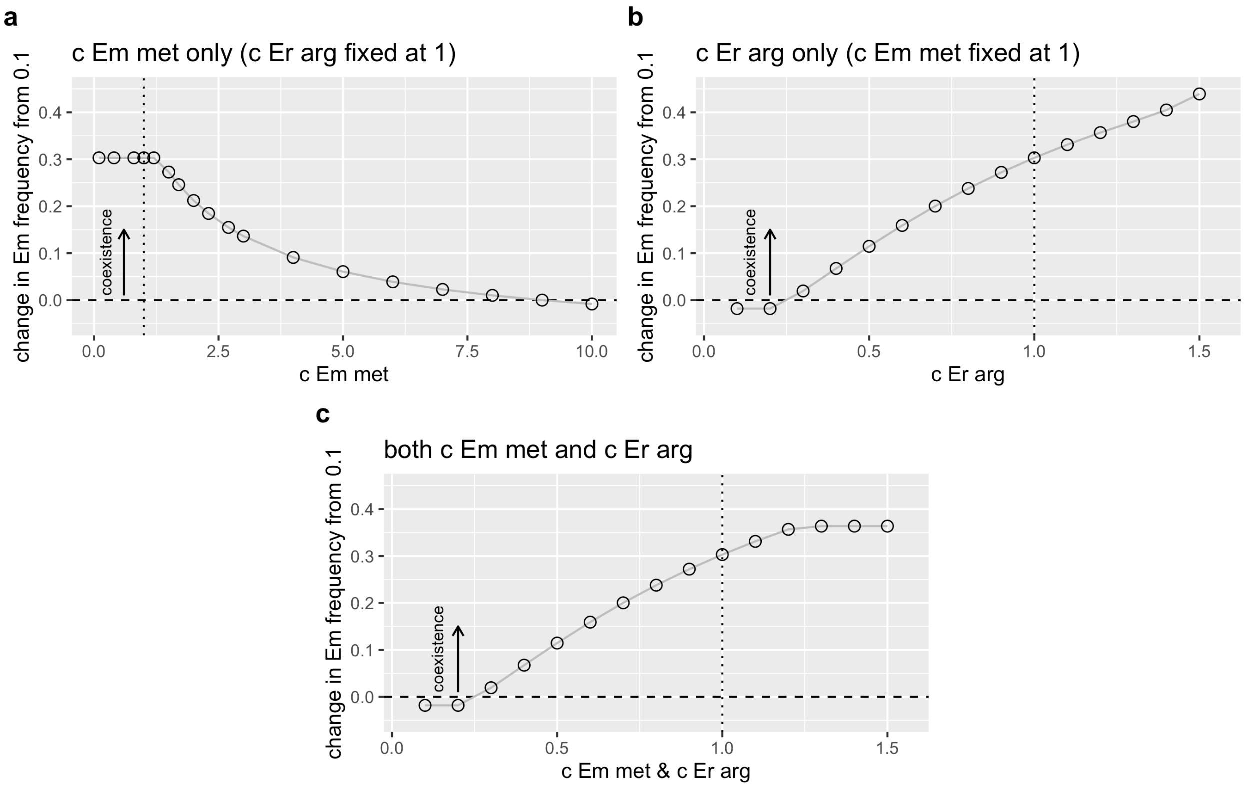

Supplementary Figure 12 | Coexistence is robust to depletion of both amino acids by both *E. coli* strains. Depletion rates $c_{Em\ arg}$ and $c_{Er\ met}$ range between 0 and 1 and adjust the rate at which each *E. coli* strain depletes the other *E. coli* strain's required amino acid. The non-required amino acid does not contribute to the population density of the strain (i.e. the equations for Em and Er are unchanged), which is supported by lab data. The default values of $c_{Em\ arg}$ and $c_{Er\ met}$ are 0. See Supplementary Table 1 for further description of these parameters and the equations below for how they are incorporated into the model. For all simulations, Em begins at a frequency of 0.1. **a**, The effect of $c_{Em\ arg}$, while $c_{Er\ met}$ is held at its default value of 0. Coexistence is maintained across the whole range from 0 to 1. **b**, The effect of $c_{Er\ met}$, while $c_{Em\ arg}$ is held at its default value of 0. Coexistence is lost at $c_{Er\ met} = 0.9$ and above. **c**, Both strains deplete the other strain's amino acid at the same rate. Between 0 and 0.9, the effects of $c_{Em\ arg}$ and $c_{Er\ met}$ cancel one another out and coexistence is maintained, but at $c_{Em\ arg} = c_{Er\ met} = 1$, coexistence is lost. For all plots, the dotted vertical line at x = 0 shows the default value of the depletion rate.

Altered equations for amino acids using these depletion rates (in bold):

$$\frac{d\,met}{dt} = -\frac{dEm}{dt} + \left(p_{Smr\ met} \times \frac{dSmr}{dt}\right) - \left(\boldsymbol{c_{Er\ met}} \times \frac{dEr}{dt}\right)$$

$$\frac{d\,arg}{dt} = -\frac{dEr}{dt} + \left(p_{Smr\ arg} \times \frac{dSmr}{dt}\right) - \left(\boldsymbol{c_{Em\ arg}} \times \frac{dEm}{dt}\right)$$

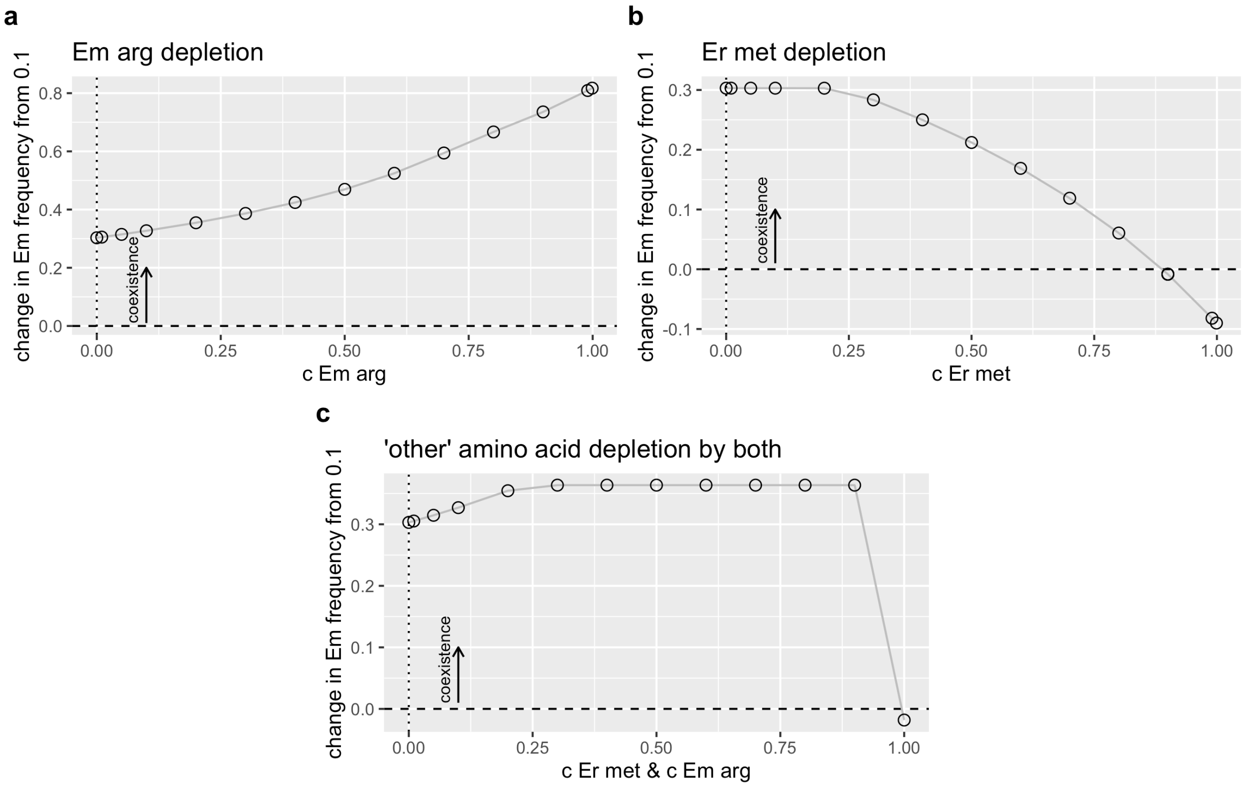

Supplementary Figure 13 | If *S. enterica* provides methionine to two different methionine-requiring *E. coli* strains, there is no coexistence, even if Sm grows more slowly. a, Schematic of the community. E met-1 (Em1) and E met- 2 (Em2) both require methionine. S met+ (Sm) produces methionine. The *E. coli* strains produce acetate, which Sm consumes, and both *E. coli* strains consume lactose. The equations are shown below. **b**, When Sm grows more slowly (μ_{Em1} = 1, μ_{Em2} = 1.1, μ_{Sm} = 0.5), there is no coexistence. The slower growing Em strain (Em1) cannot increase in frequency from any starting frequency. **c**, The same is true when Sm grows faster than the two Em strains (μ_{Em1} = 1, μ_{Em2} = 1.1, μ_{Sm} = 1.5). Em1 decreases in frequency from every starting frequency. **d**, Timeseries showing met limitation at early timepoints where μ_{Em1} = 1, μ_{Em2} = 1.1, μ_{Sm} = 0.5 and the initial frequency of Em1 is 0.1. The two *E. coli* strains compete for both methionine and lactose—methionine limits instantaneous growth rates until lactose is depleted and growth ceases.

The equations are similar to the three-strain model, except there are two *E. coli* strains that consume methionine, Em1 and Em2. Parameters are as described in Supplementary Table 1.

$$\frac{d\,Em1}{dt} = Em1 \times \mu_{Em1} \times \frac{lcts}{lcts + k_{Em1\,lcts}} \times \frac{met}{met + k_{Em1\,met}}$$

$$\frac{d\,Em2}{dt} = Em2 \times \mu_{Em2} \times \frac{lcts}{lcts + k_{Em2\,lcts}} \times \frac{met}{met + k_{Em2\,met}}$$

$$\frac{d\,Sm}{dt} = Sm \times \mu_{Sm} \times \frac{ac}{ac + k_{Sm\,ac}}$$

$$\frac{d\,lcts}{dt} = -\frac{dEm1}{dt} - \frac{dEm2}{dt}$$

$$\frac{d\,ac}{dt} = \left(p_{Em1\,ac} \times \frac{dEm1}{dt}\right) + \left(p_{Em2\,ac} \times \frac{dEm2}{dt}\right) - \frac{dSm}{dt}$$

$$\frac{d\,met}{dt} = -\frac{dEm1}{dt} - \frac{dEm2}{dt} + \left(p_{Sm\,met} \times \frac{dSm}{dt}\right)$$

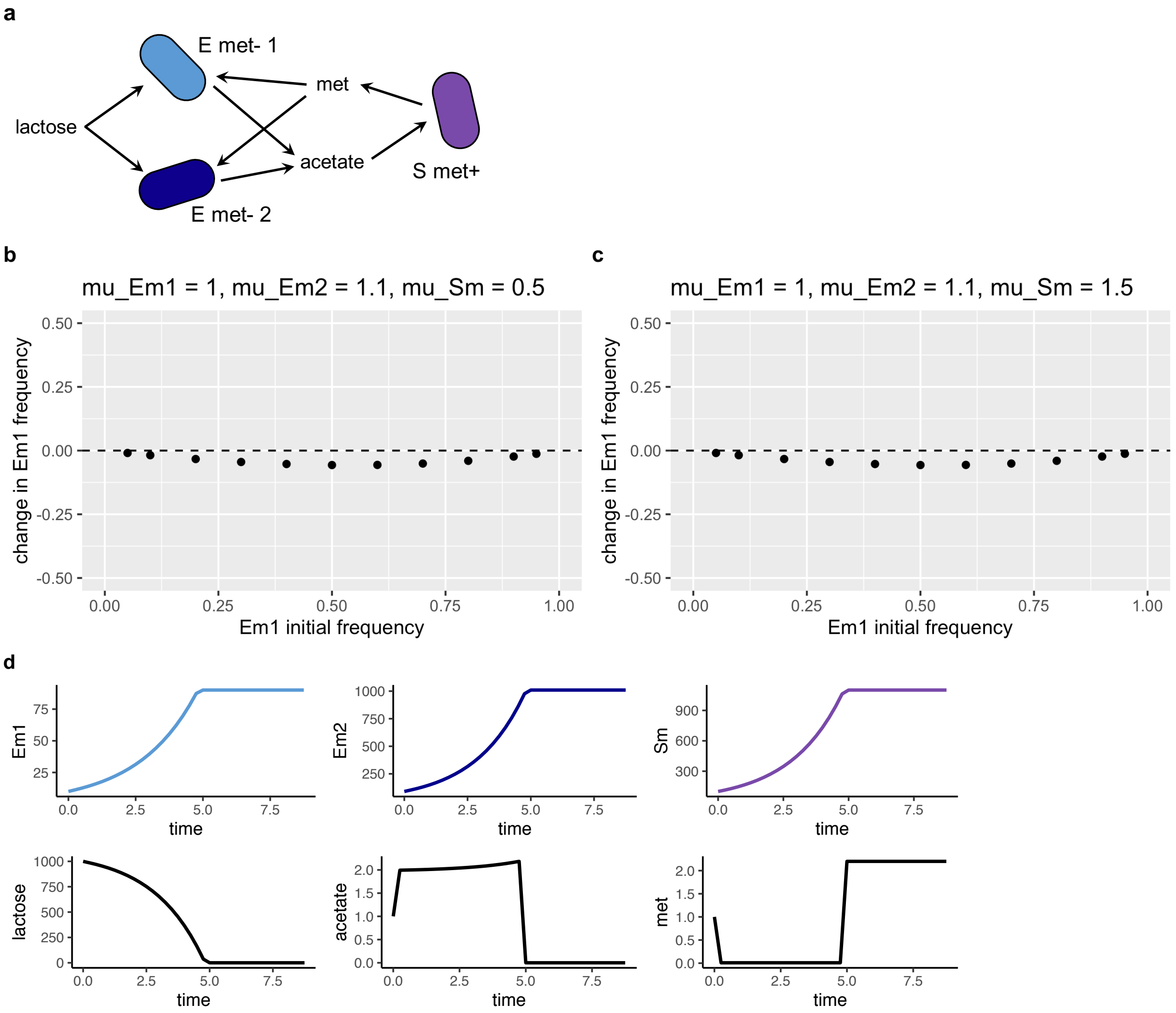
a
E met- 1
met
lactose
acetate
S met+
E met- 2
b
mu_Em1 = 1, mu_Em2 = 1.1, mu_Sm = 0.5
change in Em1 frequency
Em1 initial frequency
c
mu_Em1 = 1, mu_Em2 = 1.1, mu_Sm = 1.5
change in Em1 frequency
Em1 initial frequency
d
Em1
Em2
Sm
lactose
acetate
met
time

Supplementary Table 2 | Model parameters and default values for three *E. coli* strain community. Explanation of parameter names, units, defaults, and biological interpretations. Equations are shown below the table.

Parameter or state variable	Units	Default or initial value[1]	Name	Biological Interpretation
Smrx	Cells/ml	100 at start	Smr cell density	*S. enterica* methionine, arginine, and hypothetical amino acid x producer population size
Em	Cells/ml	variable at start	Em cell density	*E. coli* methionine auxotroph population size
Er	Cells/ml	variable at start	Er cell density	*E. coli* arginine auxotroph population size
Ex	Cells/ml	variable at start	Ex cell density	*E. coli* amino acid x auxotroph population size
lcts	Cells/ml	1000 at start	lactose	The concentration of lactose that can produce a certain cell density once consumed (i.e. cell equivalents)[2]
met	Cells/ml	1 at start	methionine	The concentration of methionine that can produce a certain cell density once consumed (i.e. cell equivalents)[2]
arg	Cells/ml	1 at start	arginine	The concentration of arginine that can produce a certain cell density once consumed (i.e. cell equivalents)[2]
x	Cells/ml	1 at start	amino acid x	The concentration of amino acid x that can produce a certain cell density once consumed (i.e. cell equivalents)[2]
ac	Cells/ml	1 at start	acetate	The concentration of acetate that can produce a certain cell density once consumed (i.e. cell equivalents)[2]
μ_{Smrx}	1/Timestep	0.5	Smr growth rate	Maximum growth rate of Smr
μ_{Em}	1/Timestep	1.0	Em growth rate	Maximum growth rate of Em
μ_{Er}	1/Timestep	1.1	Er growth rate	Maximum growth rate of Er
μ_{Ex}	1/Timestep	0.9	Ex growth rate	Maximum growth rate of Ex
$k_{Em\ lcts}$<br>$k_{Er\ lcts}$<br>$k_{Ex\ lcts}$	Cells/ml	0.01	Monod half-saturation constants	The value of k determines the resource concentration where growth rate is half-maximum

$k_{Em\ met}$<br>$k_{Er\ arg}$<br>$k_{Ex\ x}$<br>$k_{Smrx\ ac}$				
$p_{Smrx\ met}$<br>$p_{Smrx\ arg}$<br>$p_{Smrx\ x}$<br>$p_{Em\ ac}$<br>$p_{Er\ ac}$<br>$p_{Ex\ ac}$	Cells/cell	1.0001	Resource production rates	The amount of the specified resource produced by the specified strain, in cell equivalents per cell

[1] Analyses with altered parameter values are stated in the results and figure legends.

[2] Resources are defined in terms of the amount of cells that can be produced by that concentration ("cell equivalents"). Specifically, resource concentrations (mmol resource/ml) are multiplied by a conversion parameter (1 cell/mmol resource) to result in cells/ml. For example, 1000 cell equivalents of lactose means that *E. coli* would grow to a density of 1000 cells/ml.

Three *E. coli* strain model equations:

$$\frac{d\ Em}{dt} = Em \times \mu_{Em} \times \frac{lcts}{lcts + k_{Em\ lcts}} \times \frac{met}{met + k_{Em\ met}}$$

$$\frac{d\ Er}{dt} = Er \times \mu_{Er} \times \frac{lcts}{lcts + k_{Er\ lcts}} \times \frac{arg}{arg + k_{Er\ arg}}$$

$$\frac{d\ Ex}{dt} = Ex \times \mu_{Ex} \times \frac{lcts}{lcts + k_{Ex\ lcts}} \times \frac{x}{x + k_{Ex\ x}}$$

$$\frac{d\ Smrx}{dt} = Smrx \times \mu_{Smrx} \times \frac{ac}{ac + k_{Smrx\ ac}}$$

$$\frac{d\ lcts}{dt} = -\frac{dEm}{dt} - \frac{dEr}{dt} - \frac{dEx}{dt}$$

$$\frac{d\ ac}{dt} = \left(p_{Em\ ac} \times \frac{dEm}{dt}\right) + \left(p_{Er\ ac} \times \frac{dEr}{dt}\right) + \left(p_{Ex\ ac} \times \frac{dEx}{dt}\right) - \frac{dSmrx}{dt}$$

$$\frac{d\ met}{dt} = -\frac{dEm}{dt} + \left(p_{Smrx\ met} \times \frac{dSmrx}{dt}\right)$$

$$\frac{d\,arg}{dt} = -\frac{dEr}{dt} + \left(p_{Smrx\,arg} \times \frac{dSmrx}{dt}\right)$$

$$\frac{d\,x}{dt} = -\frac{dEx}{dt} + \left(p_{Smrx\,phe} \times \frac{dSmrx}{dt}\right)$$

Supplementary Figure 14 | In the three *E. coli* strain community, the rare *E. coli* strain's amino acid is abundant. Amino acid dynamics are shown for four different initial *E. coli* strain frequencies, with $\mu_{Smr} = 0.5$, $\mu_{Em} = 1$, $\mu_{Er} = 1.1$ and $\mu_{Ex} = 0.9$. **a**, Em is initially rare (at start, Em = 10, Er = 45, Ex = 45) and methionine is abundant throughout growth. **b**, Er is initially rare (at start, Em = 45, Er = 10, Ex = 45) and arginine is abundant throughout growth. **c**, Ef is initially rare (at start, Em = 45, Er = 45, Ex = 10) and amino acid x is abundant throughout growth. **d**, When two strains begin rare (at start, Em = 5, Er = 5, Ex = 90), both initially-rare strains' amino acids (methionine and amino acid x) are abundant throughout growth. The two other combinations of rare strains are not shown because dynamics are identical—the rare strains' amino acids are abundant and the common strain's amino acid is limiting.

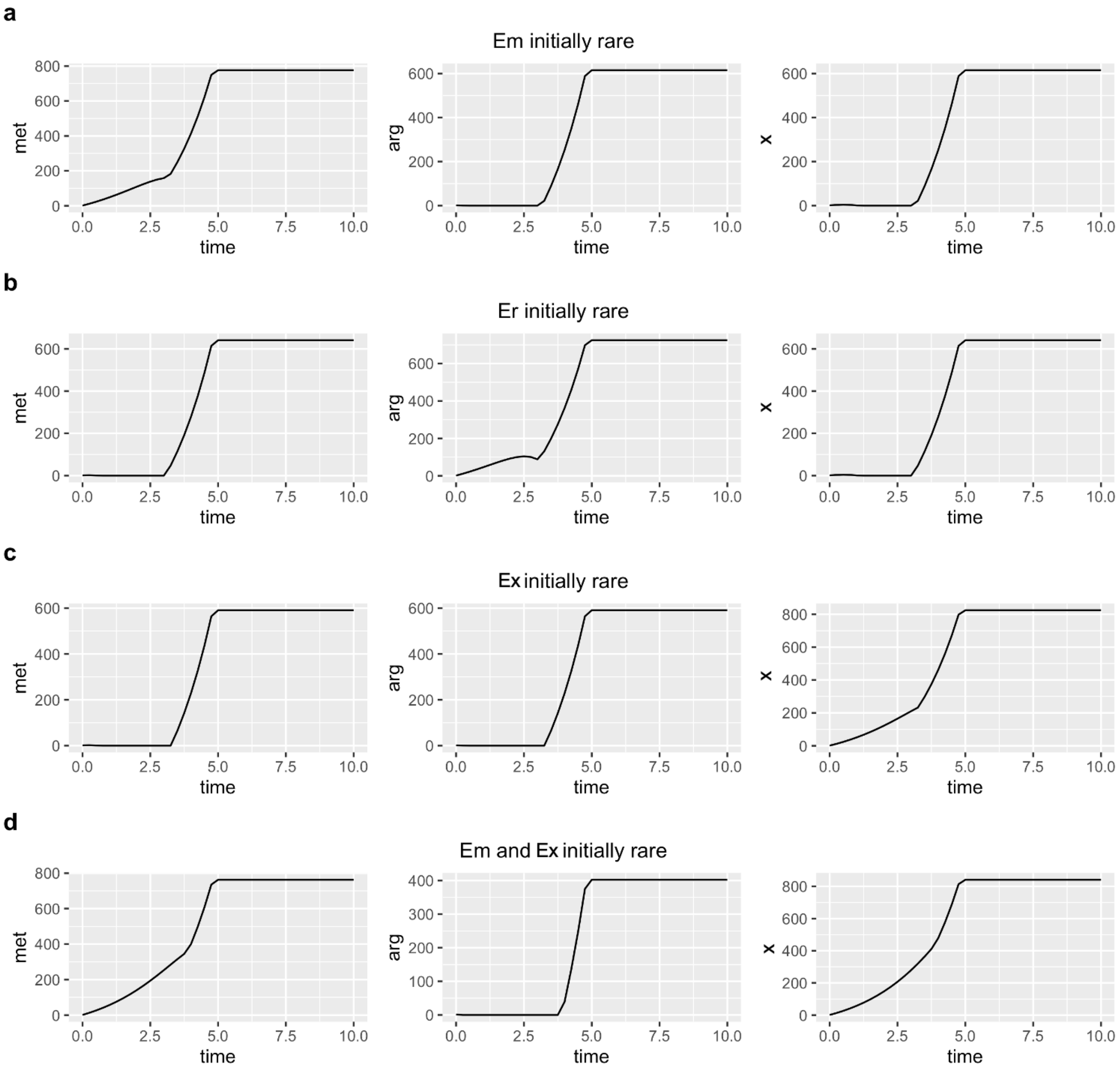

Supplementary Note 1:
Coexistence in a Chemostat

1. Chemostat Model

We consider an analogous multiple mutualist model in a chemostat with an influx rate L of lactose, and dilution rate γ.

We first summarize the results.

- There is always a stable steady state that corresponds to the washout of all microbial strains from the chemostat.
- There are two steady states that correspond to coexistence of each *E. coli* strain with the *S. enterica*, when parameters allow growth to equal dilution.
- The coexistence steady state of the *E. coli* strain with the slower growth rate (E_m) with *S. enterica*, if it exists, can be always invaded by an infinitesimal concentration of the *E. coli* strain with a faster growth rate (E_r).
- Importantly, the coexistence steady state of the *E. coli* strain with a faster growth rate (E_r) with *S. enterica*, if it exists, can be invaded by an infinitesimal concentration of the slower-growing *E. coli* strain (E_m) when the amino acid required by the slower grower is in sufficient abundance to overcome the advantage of the faster grower.
- If both 2 strain consortia of *E. coli* with *S. enterica* exist and are unstable, then the three strain consortium equilibrium exists. This result shows that in the three strain consortium chemostat model, invasibility of each two strain consortia by the third strain implies the existence of the three strain stable consortium.
- The coexistence steady state for all three strains, when it exists, induces bistability in the phase space. Some initial conditions cause communities to decrease to zero, while others converge to a stable coexistence steady state. There is also an unstable steady state whose stable manifold divides the basins of attraction of coexistence and washout steady states.
- The approach to steady state may involve multiple dramatic oscillations in the community composition. At steady state, each *E. coli* strain is co-limited by lactose and its amino acid.

We define functions

$$f_{Em}(lcts) = \frac{lcts}{lcts + k_{Em,lcts}},$$
$$f_{Er}(lcts) = \frac{lcts}{lcts + k_{Er,lcts}},$$
$$g(met) = \frac{met}{met + k_{Em,met}},$$
$$h(arg) = \frac{arg}{arg + k_{Em,arg}},$$
$$F(ac) = \frac{ac}{ac + k_{Smr,ac}}$$

and consider the following model

$$\dot{E_m} = E_m(\mu_{Em} f_{Em}(lcts) g(met) - \gamma)$$
$$\dot{E_r} = E_r(\mu_{Er} f_{Er}(lcts) h(arg) - \gamma)$$
$$\dot{Smr} = Smr(\mu_{Smr} F(ac) - \gamma)$$
$$\dot{lcts} = -E_m \mu_{Em} f_{Em}(lcts) g(met) - E_r \mu_{Er} f_{Er}(lcts) h(arg) - \gamma lact + L$$
$$\dot{ac} = p_{Em,ac} E_m \mu_{Em} f_{Em}(lcts) g(met) + p_{Er,ac} E_r \mu_{Er} f_{Er}(lcts) h(arg) - Smr \mu_{Smr} F(ac) - \gamma ac$$
$$\dot{met} = -\mu_{Em} E_m f_{Em}(lcts) g(met) + p_{Smr,met} \mu_{Smr} Smr F(ac) - \gamma met$$
$$\dot{arg} = -\mu_{Er} E_r f_{Er}(lcts) h(arg) + p_{Smr,arg} \mu_{Smr} Smr F(ac) - \gamma arg.$$

2. Parameters and assumptions

We are interested in finding steady states for this system, particularly steady states that correspond to coexistence of a single strain of *E. coli* with *S. enterica* at the steady state that corresponds to coexistence of all three strains.

In order to facilitate our analysis, we simplify the notation using parameter choices that were used to simulate batch experiments:

$$\begin{aligned} k_{Em,lcts} &= k_{Er,lcts} = k_{Em,met} = k_{Em,arg} = k_{Smr,ac} = 0.01 \\ p_{Em,ac} &= p_{Er,ac} = p_{Smr,met} = p_{Smr,arg} = 1.0001 \\ \mu_{Smr} &= 0.5; \\ \mu_{Er} &= 1.1; \\ \mu_{Em} &= 1. \end{aligned} \tag{1}$$

In particular we will make the following assumptions:

(a): Since the half-saturation constants are the same, all the Hill functions in the problem are identical, only evaluated at different substrates. Therefore we write

$$f(x) := f_{Em}(x) = f_{Er}(x) = g(x) = h(x) = F(x).$$

This assumption is not essential to our arguments about the existence of various steady states as our arguments only depend on the monotonicity of the Hill functions.

(b): We assume that the production rates of amino acids by *S. enterica* are the same

$$p := p_{Smr,arg} = p_{Smr,met}.$$

(c): We assume that $\mu_{Er} > \mu_{Em}$. The opposite assumption will lead to analogous results with *E. coli* strain identity exchanged.

Further, we use a shortened version of the variables above and write S for Smr, ℓ for $lcts$, c for ac, m for met and a for arg. Then the system becomes

$$\begin{aligned}
\dot{E}_m &= E_m(\mu_{Em} f(\ell) f(m) - \gamma) \\
\dot{E}_r &= E_r(\mu_{Er} f(\ell) f(a) - \gamma) \\
\dot{S} &= S(\mu_{Smr} f(c) - \gamma) \\
\dot{\ell} &= -E_m \mu_{Em} f(\ell) f(m) - E_r \mu_{Er} f(\ell) f(a) - \gamma \ell + L \\
\dot{c} &= p_{Em,ac} E_m \mu_{Em} f(\ell) f(m) + p_{Er,ac} E_r \mu_{Er} f(\ell) f(a) - S \mu_{Smr} f(c) - \gamma c \\
\dot{m} &= -\mu_{Em} E_m f(\ell) f(m) + p \mu_{Smr} S f(c) - \gamma m \\
\dot{a} &= -\mu_{Er} E_r f(\ell) f(a) + p \mu_{Smr} S f(c) - \gamma a.
\end{aligned} \tag{2}$$

3. Steady states

In this section, we compute the steady states of the chemostat. Our analysis will focus on the existence of different coexistence steady states and their stability. Where we can, we will make general statements valid for all parameters, and where we cannot make general statements, we will provide formulas in terms of parameters. Our last resort will be to provide numerical support for our assertions for the parameters that we use for the model.

The steady state equations are

$$\begin{aligned}
0 &= E_m(\mu_{Em} f(\ell) f(m) - \gamma) \\
0 &= E_r(\mu_{Er} f(\ell) f(a) - \gamma) \\
0 &= S(\mu_{Smr} f(c) - \gamma) \\
0 &= -E_m \mu_{Em} f(\ell) f(m) - E_r \mu_{Er} f(\ell) f(a) - \gamma \ell + L \\
0 &= p_{Em,ac} E_m \mu_{Em} f(\ell) f(m) + p_{Er,ac} E_r \mu_{Er} f(\ell) f(a) - S \mu_{Smr} f(c) - \gamma c \\
0 &= -\mu_{Em} E_m f(\ell) f(m) + p \mu_{Smr} S f(c) - \gamma m \\
0 &= -\mu_{Er} E_r f(\ell) f(a) + p \mu_{Smr} S f(c) - \gamma a.
\end{aligned} \tag{3}$$

3.1. **Trivial steady state.** We have the following result for the trivial steady state, where all microbial strains wash out of the chemostat.

Theorem 3.1. *In the absence of S. enterica, i.e when $S = 0$, the only steady state is a trivial steady state with $E_m = 0, E_r = 0$. At this steady state, the concentration of amino acids and acetate are zero $c = 0, a = 0, m = 0$ and lactose is given by the influx and dilution rates of the chemostat $\ell = \frac{L}{\gamma}$.*

Proof. Assume that $S = 0$ so there is no *S. enterica* present. Then from the last two equations in (3) we get $m = 0$ and $a = 0$. This forces the first two equations to have $E_m = E_r = 0$, which then forces $c = 0$ and, finally, $\ell = \frac{L}{\gamma}$. This is the trivial steady state. □

Theorem 3.2. *The trivial steady state is asymptotically stable i.e. all solutions starting nearby will converge to it.*

This proof is delegated to section 4.

3.2. **Coexistence steady states.** Now we will investigate existence of steady states where some of the microbial strains coexist. We start analysis by looking for a steady state where all three strains are present i.e. $E_m > 0, S > 0, E_r > 0$. With this assumption, it follows from the first three equations in (3) that

$$\mu_{Em} f(\ell) f(m) = \gamma \tag{4}$$

$$\mu_{Er} f(\ell) f(a) = \gamma \tag{5}$$

$$\mu_{Smr} f(c) = \gamma. \tag{6}$$

Using these relations in the last four equations in (3) we get

$$\begin{aligned} \ell &= -(E_m + E_r) + \frac{L}{\gamma} \\ c &= p_{Em,ac} E_m + p_{Er,ac} E_r - S \\ m &= -E_m + pS \\ a &= -E_r + pS. \end{aligned} \tag{7}$$

We first solve the equation (6). Since the function f is increasing, there is a unique steady state value of acetate, which we denote c^*, which satisfies

$$\mu_{Smr} f(c^*) = \gamma.$$

From now on, we will denote steady state concentrations by asterisk *. From the second equation in (7) we obtain a restriction on the steady state balance between consumption of acetate and the production of acetate $c^* = p_{Em,ac} E_m^* + p_{Er,ac} E_r^* - S^*$. We express the steady state concentration S^* as a function of steady state concentrations of both *E. coli*

$$S^* = p_{Em,ac} E_m^* + p_{Er,ac} E_r^* - c^*. \tag{8}$$

This population of *S. enterica* produces steady state value of amino acids

$$m^* = -E_m^* + p(p_{Em,ac} E_m^* + p_{Er,ac} E_r^* - c^*) \tag{9}$$

$$a^* = -E_r^* + p(p_{Em,ac} E_m^* + p_{Er,ac} E_r^* - c^*) \tag{10}$$

The steady state value of lactose is also expressed as a function of E_m^*, E_r^*

$$\ell^* = \frac{L}{\gamma} - (E_m^* + E_r^*). \tag{11}$$

Any steady state of the system (2) has to satisfy equations (8,9,10,11) with population concentrations $E_m^* \geq 0, E_r^* \geq 0$. The additional constraints come from the equations (4,5,6).

We now discuss different types of coexistence steady states.

3.2.1. *2-strain coexistence steady states.* We first discuss conditions for existence of a steady state Q_m with

$$E_m > 0, S > 0, E_r = 0.$$

Only one strain of *E. coli* is present in a mutualistic coexistence with *S. enterica*.

Theorem 3.3. *The steady state Q_m, if it exists, is always unstable.*

Proof. Since $E_m > 0$ the equation (4) is valid at the steady state Q_m, which states that the growth rate of E_m must be equal to the dilution rate of the chemostat. Plugging expressions equations (11) and (9) to (4) with $E_r = 0$ we get

$$\mu_{Em} f(\frac{L}{\gamma} - E_m^*) f(-E_m^* + p(p_{Er,ac} E_m^* - c^*)) = \gamma. \tag{12}$$

This is a single equation in E_m^* which may or may not have a positive solution. Note that the argument of the first function is the steady state amount of lactose that decreases with E_m^* and the argument of the second function is the steady state concentration of methionine. If these values are sufficiently high to produce a growth rate for E_m that matches the dilution rate, two strain coexistence steady state is possible.

If steady state Q_m exist, then at Q_m the following holds

$$\begin{aligned} \mu_{Er} f(\ell^*) f(a^*) &= \mu_{Er} f(\frac{L}{\gamma} - E_m^*) f(p(p_{Er,ac} E_m^* - c^*)) \\ &> \mu_{Em} f(\frac{L}{\gamma} - E_m^*) f(p(p_{Er,ac} E_m^* - c^*)) \\ &> \mu_{Em} f(\frac{L}{\gamma} - E_m^*) f(-E_m^* + p(p_{Er,ac} E_m^* - c^*)) \\ &= \gamma. \end{aligned} \tag{13}$$

We show in Lemma 4.1 that one of the eigenvalues of Jacobian $J(Q_m)$ is

$$\lambda = \mu_{Er} f(\ell^*) f(a^*) - \gamma.$$

By (13) it follows that $\lambda > 0$ and therefore Q_m, if it exist, is always unstable.

Remark 3.4. The positivity of eigenvalue λ that implies instability of Q_m is equivalent to a statement that the steady state Q_m can be invaded by an infinitesimal population of E_r.

Remark 3.5. Note that the argument in (13) that shows $\lambda > 0$ depends on two inequalities. The first inequality is between the growth rates $\mu_{Er} > \mu_{Em}$; it is not surprising that the strain with the faster specific growth rate may invade the consortium of the slower-growing *E. coli.* Importantly, the second inequality is based on the fact that the concentration of available arginine is greater that the concentration of available methionine, because the methionine is being consumed by E_m, but arginine, while being produced by *S. enterica*, is not being utilized at Q_m where $E_r^* = 0$. Therefore production of arginine by *S. enterica* is creating a niche that can be exploited by invading

E_r. Crucially, as we show below, this type of niche created by the faster-growing E_r can be also exploited by invading *E. coli* E_m with a slower specific growth rate.

We now discuss conditions for the existence of a steady state Q_r with

$$E_r > 0, S > 0, E_m = 0.$$

The argument is completely analogous with one important distinction. Note that only the first inequality in (13) depends on $\mu_{Er} > \mu_{Em}$, but the second does not. This means that it is possible that if the growth rates of two *E. coli* strains are near each other, as is the case in the *E. coli* strains considered in this paper, that the slower-growing strain E_m will invade steady state Q_r.

This leads to the following result:

Theorem 3.6. *Assume that at the steady state Q_r, if it exists, we have*

$$\mu_{Em} f(m^*) > \mu_{Er} f(a^*). \tag{14}$$

Then Q_r is unstable and infinitesimal population of E_m can invade Q_r.

Proof. The argument is analogous to the proof of Theorem 3.3. The key argument, analogous to (13) is as follows

$$\mu_{Em} f(\ell^*) f(m^*) > \mu_{Er} f(\ell^*) f(a^*) = \gamma. \tag{15}$$

The inequality is equivalent to the assumption (14), since the equality follows from the the equation (4) that holds at Q_r.

The instability now follows from Lemma 4.1 that shows that one of the eigenvalues of Jacobian $J(Q_r)$ is

$$\mu = \mu_{Em} f(\ell^*) f(m^*) - \gamma.$$

Remark 3.7. Assumption (14) states that if the ratio of m^* to $a/^*$ is larger than μ_{Em}/μ_{Er} then the slower E_m can invade a consortium of the faster E_r paired with *S. enterica*

3.3. **Coexistence of 3 strains.** Consider the three strain steady state C (for 'coexistence')

$$E_m \neq 0, S \neq 0, E_r \neq 0.$$

The equations (4,5) together with (9,10,11) give

$$\mu_{Em} f(\frac{L}{\gamma} - E_m^* - E_r^*) f(-E_m^* + p(p_{Em,ac} E_m^* + p_{Er,ac} E_r^* - c^*) = \gamma$$

$$\mu_{Er} f(\frac{L}{\gamma} - E_m^* - E_r^*) f(-E_r^* + p(p_{Em,ac} E_m^* + p_{Er,ac} E_r^* - c^*)) = \gamma. \tag{16}$$

This system of nonlinear equations in E_m^*, E_r^* may or may not have a positive solution. This depends on the values of parameters and we were unable to find general relationships between the parameters that would guarantee existence of such a solution. However, we show that if Q_r, Q_m exist and are both unstable, the coexistence steady state must exist.

Theorem 3.8. *Assume that both Q_r and Q_m two strain consortia are feasible. Then under the assumption (14) which guarantees invasibility of Q_r by E_m, there is a coexistence steady state C where all three strains coexist.*

Proof. In order to show existence of C, we need to show that there are values E_m^* and E_r^* that satisfy the pair of nonlinear equations (16). To simplify the notation and more clearly show the symmetry in the two equations (16), we start by defining a function

$$h(x,y) := f(\frac{L}{\gamma} - x - y)f(-x + p(px + py - c^*)), \tag{17}$$

where we used assumption that $p = p_{Em,ac} = p_{Er,ac}$ and replaced E_m, E_r by x, y. Then the condition (16) for the existence of the coexistence equilibrium C is existence of values

$$x^* = E_m^* \quad \text{and} \quad y^* = E_r^*$$

such that

$$h(x^*, y^*) = \frac{\gamma}{\mu_{Em}} \quad \text{and} \quad h(y^*, x^*) = \frac{\gamma}{\mu_{Er}}. \tag{18}$$

Note that there is the change of the two arguments of the function h, which expresses symmetry between the strains E_m and E_r. At the same time, the different values on the right hand side are the consequence of different growth rates of E_m and E_r.

The existence of the 2 strain steady state Q_m is equivalent to solving (12), which in the language of the function $h(x,y)$ can be expressed as the existence of $x_0 > 0$ such that $h(x_0, 0) = \frac{\gamma}{\mu_{Em}}$. Therefore, the existence of Q_m and Q_r are equivalent to existence of y_0, x_0 that satisfy, respectively,

$$h(x_0, 0) = \frac{\gamma}{\mu_{Em}} \quad \text{and} \quad h(y_0, 0) = \frac{\gamma}{\mu_{Er}}. \tag{19}$$

By direct computation, the second derivative $\frac{d^2h(u,0)}{du^2} < 0$ and $\frac{d^2h(0,v)}{dv^2} < 0$ which means that the function h is concave down in both variables and has a maximum. The existence of the 2 strain consortia (19) guarantees the existence of solutions of $h(x,0) = \alpha$ for two values of $\alpha = \frac{\gamma}{\mu_{Em}}$ and $\alpha = \frac{\gamma}{\mu_{Er}}$. However, the concavity shows that there must be (generically) two solutions for each constant. In particular, there are four points on the x-axis, ordered as in Figure 15 such that

$$h(x_0^1, 0) = h(x_0^2, 0) = \frac{\gamma}{\mu_{Em}}, \qquad \text{and} \qquad h(y_0^1, 0) = h(y_0^2, 0) = \frac{\gamma}{\mu_{Er}}.$$

The order of the points reflects the assumption that $\mu_{Em} < \mu_{Er}$. The same points are also marked on the y-axis.

Next, we examine the function $h(x,y)$ along the lines $x + y = C$, where C is a constant. This function has the form

$$g_C(x) := f(\frac{L}{\gamma} - C)f(-x + p(pC - c^*)).$$

Since f is an increasing function of its argument, $g_C(x)$ is a decreasing function of x for any choice of C. Therefore along the line segment connecting points $(0, y_0^1)$ and $(y_0^1, 0)$, that is the along the line segment, $x + y = y_0^1$ the value of the function $h(x,y)$ decreases with x. Consequently, the level curve of $h(x,y) = \frac{\gamma}{\mu_{Er}}$ that starts at the point $(y_0^1, 0)$ must terminate on the y-axis at a point with y-coordinate below y_0^1. The same argument, but taking into account that the maximum of $h(x,0)$ occurs on the x-axis between x_0^1 and x_0^2, shows that the level curve of $h(x,y) = \frac{\gamma}{\mu_{Er}}$ that starts at the point $(y_0^2, 0)$ must terminate on the y-axis at a point with y-coordinate above y_0^2. Both of these level curves are denoted by dashed lines in Figure 15. A symmetric argument applies to red curves

that represent the level curves of $h(y,x) = \frac{\gamma}{\mu_{Er}}$, starting at $(0, y_0^1)$ and $(0, y_0^2)$ on the y-axis. Note that by symmetry red and red dotted curves intersect at $x = y$ axis.

Our final piece of the information comes from (15), which is equivalent to (14) that guarantees invasibility of the 2 strain consortium Q_r by the slower grower E_m:

$$f(\ell^*)f(m^*) > \frac{\gamma}{\mu_{Em}}.$$

Writing out the details on left hand side we get

$$f(\frac{L}{\gamma} - E_r^*)f(p(pE_r^* - c^*) > \frac{\gamma}{\mu_{Em}}.$$

We compare the left hand side with the definition of the function $h(x,y)$ (17), this is equivalent (with $y_0 = E_r^*$)

$$h(0, y_0) = f(\frac{L}{\gamma} - y_0)f(p(py_0 - c^*)).$$

Therefore the assumption (14) on invasibility of Q_m implies

$$h(0, y_0) > \frac{\gamma}{\mu_{Em}}. \tag{20}$$

Consider two branches of the level set $h(x,y) = \frac{\gamma}{\mu_{Em}}$ that start at x_0^1 and x_0^2 on the x-axis, respectively (blue lines in Figure 15). The statement (20) shows that the lower branch terminates bellow y_0^1 and the upper branch terminates above y_0^2 on the y-axis.

The rest of the proof relies on two dimensional geometry in the plane. Figure 15 assembles the information derived above about the shapes of the level curves of $h(x,y) = \frac{\gamma}{\mu_{Er}}$ (dashed), $h(x,y) = \frac{\gamma}{\mu_{Em}}$ (blue) and $h(y,x) = \frac{\gamma}{\mu_{Er}}$ (red). The conclusion from the Figure 15 is that the red and blue curves must have at least two intersections, as indicated. These coordinates of intersections x^*, y^* satisfy

$$h(x^*, y^*) = \frac{\gamma}{\mu_{Er}} \qquad \text{and} \qquad h(y^*, x^*) = \frac{\gamma}{\mu_{Em}}.$$

which by (18) is equivalent to existence of 3 strain co-existence solutions.

Numerical simulations in Section 5 suggest that the coexistence equilibrium S is a saddle point and the coexistence equilibrium C is stable. This, in conjunction with a stable trivial equilibrium, supports the bistable picture of the coexistence dynamics depicted in Figure 16.

4. Stability of the equilibria.

To determine stability of the equilibria, we compute a Jacobian J of the system (2):

$$\begin{bmatrix}
\mu_m f(\ell)f(m) - \gamma & 0 & 0 & E_m\mu_m f(m)f'(\ell) & 0 & E_m\mu_m f'(m)f(\ell) & 0 \\
0 & \mu_r f(\ell)f(a) - \gamma & 0 & E_r\mu_r f(a)f'(\ell) & 0 & 0 & E_r\mu_r f'(a)f(\ell) \\
0 & 0 & \mu_S f(c) - \gamma & 0 & S\mu_S f'(c) & 0 & 0 \\
-\mu_m f(\ell)f(m) & -\mu_r f(\ell)f(a) & 0 & A & 0 & -E_m\mu_m f'(m)f(\ell) & -E_r\mu_r f'(a)f(\ell) \\
p_m\mu_m f(\ell)g(m) & p_r\mu_r f(\ell)h(a) & \mu_S f(c) & B & -\mu_S Sf'(c) - \gamma & p_m E_m\mu_m f'(m)f(\ell) & p_r E_r\mu_r f'(a)f(\ell) \\
-\mu_m f(\ell)f(m) & 0 & p\mu_S f(c) & -E_m\mu_m f(m)f'(\ell) & p\mu_S Sf'(c) & -E_m\mu_m f'(m)f(\ell) - \gamma & 0 \\
0 & -\mu_r f(\ell)f(a) & p\mu_S f(c) & -E_r\mu_r f(a)f'(\ell) & p\mu_S Sf'(c) & 0 & -E_r\mu_r f'(a)f(\ell) - \gamma
\end{bmatrix}.$$

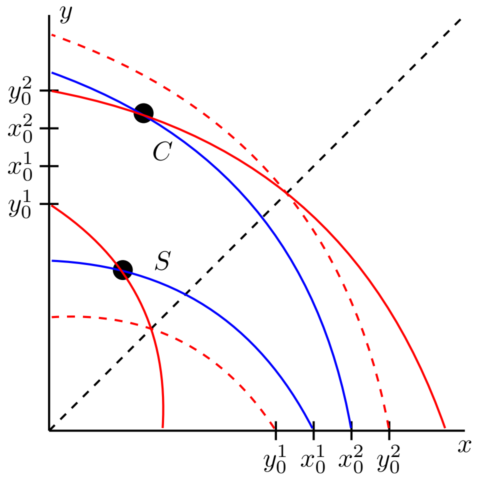

FIGURE 15. The blue curves are the level curve $h(x, y) = \frac{\gamma}{E_m}$ and the red curves are level curves of $h(y, x) = \frac{\gamma}{E_r}$. Their intersections S and C represents solutions of (16) and hence 3-strain coexistence solutions. The intersections of these curves with the x-axis, $x_0^1, x_0^2 y_0^1, y_0^2$ follow from the existence of coexistence equilibria Q_r and Q_m. The same values are marked on the y-axis. The consequence of (20) is that the blue level curves intersect the y-axis bellow and above the values x_0^1 and x_0^2. respectively.

where we use the following notation in order to fit the matrix to the page

$$\begin{aligned} A &:= -f'(\ell)(E_m \mu_m f(m) + E_r \mu_r f(a)) - \gamma \\ B &:= f'(\ell)(p_m E_m \mu_m f(m) + p_r E_r \mu_r f(a)) \\ p_m &= p_{Em,ac}, p_r = p_{Er,ac}, \\ \mu_m &= \mu_{Em}, \mu_r = \mu_{Er}, \mu_S = \mu_{Smr}. \end{aligned}$$

Proof of Theorem 3.2

We evaluate the Jacobian at the trivial steady state to get

$$\begin{bmatrix} -\gamma & 0 & 0 & 0 & 0 & 0 & 0 \\ 0 & -\gamma & 0 & & 0 & 0 & 0 \\ 0 & 0 & -\gamma & 0 & 0 & 0 & 0 \\ 0 & 0 & 0 & -\gamma & 0 & 0 & 0 \\ 0 & 0 & 0 & 0 & -\gamma & 0 & 0 \\ 0 & 0 & 0 & 0 & 0 & -\gamma & 0 \\ 0 & 0 & 0 & 0 & 0 & 0 & -\gamma \end{bmatrix}.$$

This matrix has 7 eigenvalues with negative real parts and hence is asymptotically stable.

Lemma 4.1. *The Jacobian evaluated at the two strain steady state Q_r has an eigenvalue*

$$\mu = \mu_{Em} f(\ell^*) f(m^*) - \gamma.$$

The Jacobian evaluated at the two strain steady state Q_m has an eigenvalue

$$\lambda = \mu_{Er} f(\ell^*) f(a^*) - \gamma$$

Proof. We evaluate the Jacobian at an steady state Q_r with $E_m = 0, S > 0, E_r > 0$ which implies

$$\mu_{Smr} f(c) = \gamma \quad \text{and} \quad \mu_{Er} f(\ell) f(a) = \gamma.$$

The Jacobian becomes

$$\begin{bmatrix} \mu_{Em} f(\ell) f(m) - \gamma & 0 & 0 & 0 & 0 & 0 & 0 \\ 0 & 0 & 0 & E_r \mu_{Er} f(a) f'(\ell) & 0 & 0 & E_r \mu_{Er} f'(a) f(\ell) \\ 0 & 0 & 0 & 0 & S \mu_{Smr} f'(c) & 0 & 0 \\ -\mu_{Em} f(\ell) f(m) & -\gamma & 0 & -f'(\ell) E_r \mu_{Er} f(a) - \gamma & 0 & 0 & -E_r \mu_{Er} f'(a) f(\ell) \\ p_m \mu_{Em} f(\ell) f(m) & p_r \gamma & -\gamma & f'(\ell) p_r E_r \mu_{Er} f(a)) & -\mu_{Smr} S f'(c) - \gamma & 0 & p_r E_r \mu_{Er} f'(a) f(\ell) \\ -\mu_{Em} f(\ell) f(m) & 0 & p\gamma & 0 & p \mu_{Smr} S f'(c) & -\gamma & 0 \\ 0 & -\mu_{Er} f(\ell) f(a) & p\gamma & -E_r \mu_{Er} f(a) f'(\ell) & p \mu_{Smr} S f'(c) & 0 & -E_r \mu_{Er} f'(a) f(\ell) - \gamma \end{bmatrix}.$$

We note that by expanding the Jacobian along the first row, one of the eigenvalues is

$$\mu = \mu_{Em} f(\ell^*) f(m^*) - \gamma.$$

An analogous argument shows that the Jacobian at Q_m, where

$$\mu_{Smr} f(c) = \gamma \quad \text{and} \quad \mu_{Em} f(\ell) f(m) = \gamma$$

has an eigenvalue

$$\lambda = \mu_{Er} f(\ell^*) f(a^*) - \gamma.$$

5. Numerical simulations

We illustrate the existence of three strain coexistence steady states, where both strains of *E. coli* coexist with *S. enterica*, by numerical simulation using the same parameters that were used in the batch model. Since we are using a chemostat model, there are two new parameters L, which represents the concentration of lactose in the influx to the chemostat, and the dilution rate γ. For the simulations we will use parameters 1 and

$$L = 0.8, \qquad \gamma = 0.4.$$

The simulations show that there are two coexistence steady states, one of which is locally asymptotically stable, denoted C for "coexistence", and the other is a saddle point, denoted S.

$$S := (E_r, E_m, Smr, lcts, ac, met, arg) = (0.3198, 0.3189, 0.3256, 1.6345, 0.3132, 0.0058, 0.0067)$$
$$C := (E_r, E_m, Smr, lcts, ac, met, arg) = (1.8571, 0.1362, 1.9535, 0.0067, 0.0400, 0.0966, 1.8175)$$

The saddle point is unstable with a one-dimensional unstable manifold. The stability properties have been confirmed by evaluating Jacobian at C and S and computing eigenvalues.

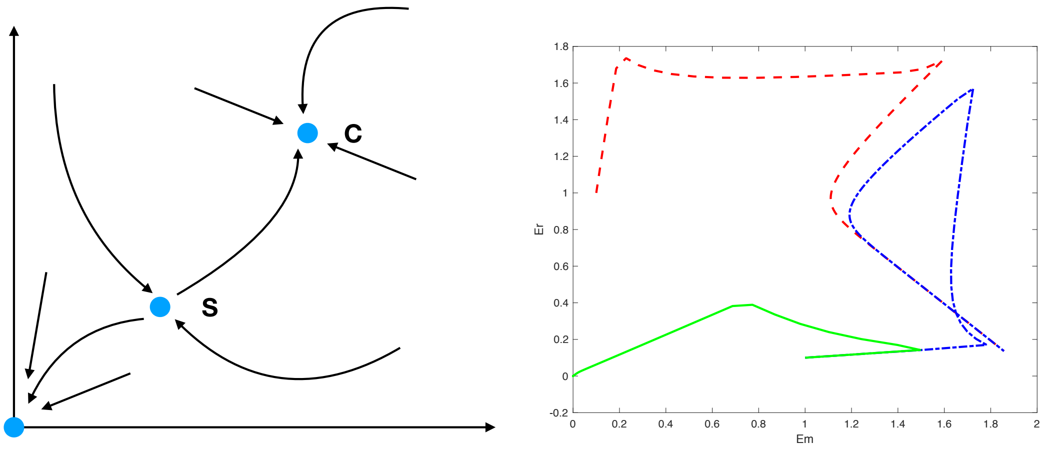

FIGURE 16. (Left) Conceptual picture of the 7-dimensional phase space. The trivial steady state is stable. If the coexistence steady states exist, then S is a saddle and C is stable. (Right) Projection of three trajectories in $\mathbf{R}^7$ to into $\mathbf{R}^2$ of concentrations of two *E. coli* strain. The red dashed curve with initial condition $(E_r, E_m, Smr, lcts, ac, met, arg) = (1, 0.1, 1, 10, 1, 1, 1)$ and the blue dashed-dot curve initial condition $(0.1, 1, 1, 10, 1, 1, 1)$ both converge to the coexistence steady state C in the lower right of the Figure. The green curve starts at the same values of E_m, E_r as the blue curve, but with a small initial concentration of *S. enterica* $(1, 0.1, 0.01, 10, 1, 1, 1)$; this population converges to the trivial steady state and does not survive.

We summarize our observations in Figure 17. In the left panel, we show a conceptual picture in the phase space $\mathbf{R}^7$, which shows bistability between the trivial washout steady state and the coexistence steady state C. Basins of attractions of these points are separated by six dimensional stable manifold of the saddle point S. Therefore, which steady state the population will reach will depend on the initial population and the initial distribution of resources. In the right panel, we project three trajectories starting at three different initial condition to two dimensional space of E_m, E_r concentrations. The red dashed line with initial condition

$$(E_r, E_m, Smr, lcts, ac, met, arg) = (1, 0.1, 1, 10, 1, 1, 1)$$

and blue dashed-dot line initial condition

$$(E_r, E_m, Smr, lcts, ac, met, arg) = (0.1, 1, 1, 10, 1, 1, 1)$$

both converge to coexistence steady state C in the lower right corner of the figure. The green curve starts at the same values of E_m, E_r as the blue curve, but with a small initial concentration of *S. enterica*

$$(E_r, E_m, Smr, lcts, ac, met, arg) = (1, 0.1, 0.01, 10, 1, 1, 1);$$

this population converges to the trivial steady state and does not survive.

We illustrate the dynamics changes in the consortium composition along the way to coexistence steady state C in Figure 17.We start with initial data

$$(E_r, E_m, Smr, lcts, ac, met, arg) = (1, 1, 1, 1000, 1, 1, 1)$$

and graph the behavior of all microbial strains and resources, except lactose. The lactose initial concentration is high to match the batch experiments and its inclusion skews the axis in a way that obscures the dynamic periodic changes in the population dynamics. We observe fast exponential growth in the E_r and E_m strains until both methionine and arginine are exhausted; the acetate that the *E. coli* produced allows the slower-growing *S. enterica* to continue to grow and thus replenish the amino acid pools in the chemostat. This induces a rebound in the *E. coli* strain around time 12. The exhaustion of the acetate pool leads to decline of the *S. enterica* population toward the steady state, starting around the time 15. This decline induces a second decline in *E. coli* populations, which then slowly converge to their steady state values. Note that the steady state value of E_r is higher than that of E_m, reflecting the difference in higher specific growth rates between these strains.

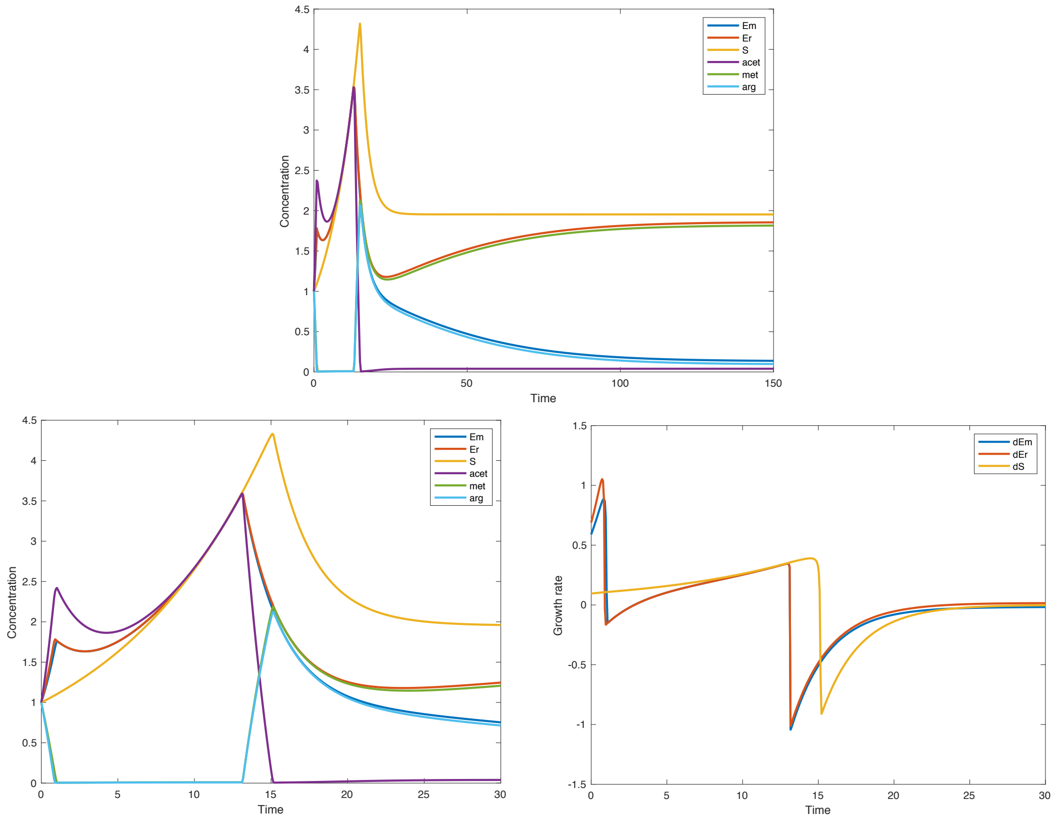

FIGURE 17. (Top) Concentrations converge to the stable steady state C for the parameters listed above. (Lower Left) Detail of the concentrations for first 30 units of time. (Lower Right) Growth rates of the *E. coli* strains and *S. enterica* show rapid changes along the way to the steady state. Initial data for all three figures $(E_r, E_m, Smr, lcts, ac, met, arg) = (1, 1, 1, 1000, 1, 1, 1)$.
