# Supplementary material for: Limitation by a shared mutualist promotes coexistence of multiple competing partners
Source: Nat Commun. 2021 Jan 27;12:619. doi: 10.1038/s41467-021-20922-0 (PMC7840915; doi:10.1038/s41467-021-20922-0)
Supplement: Supplementary file 3 — Reporting Summary [file 41467_2021_20922_MOESM3_ESM.pdf]

nature research

Corresponding author(s): William Harcombe

Last updated by author(s): Dec 14, 2020

Reporting Summary

Nature Research wishes to improve the reproducibility of the work that we publish. This form provides structure for consistency and transparency in reporting. For further information on Nature Research policies, see our Editorial Policies and the Editorial Policy Checklist.

Statistics

For all statistical analyses, confirm that the following items are present in the figure legend, table legend, main text, or Methods section.

n/a	Confirmed	
☐	☒	The exact sample size (*n*) for each experimental group/condition, given as a discrete number and unit of measurement
☐	☒	A statement on whether measurements were taken from distinct samples or whether the same sample was measured repeatedly
☐	☒	The statistical test(s) used AND whether they are one- or two-sided<br>*Only common tests should be described solely by name; describe more complex techniques in the Methods section.*
☒	☐	A description of all covariates tested
☐	☒	A description of any assumptions or corrections, such as tests of normality and adjustment for multiple comparisons
☐	☒	A full description of the statistical parameters including central tendency (e.g. means) or other basic estimates (e.g. regression coefficient) AND variation (e.g. standard deviation) or associated estimates of uncertainty (e.g. confidence intervals)
☐	☒	For null hypothesis testing, the test statistic (e.g. *F*, *t*, *r*) with confidence intervals, effect sizes, degrees of freedom and *P* value noted<br>*Give P values as exact values whenever suitable.*
☒	☐	For Bayesian analysis, information on the choice of priors and Markov chain Monte Carlo settings
☒	☐	For hierarchical and complex designs, identification of the appropriate level for tests and full reporting of outcomes
☒	☐	Estimates of effect sizes (e.g. Cohen's *d*, Pearson's *r*), indicating how they were calculated

Our web collection on statistics for biologists contains articles on many of the points above.

Software and code

Policy information about availability of computer code

Data collection	Modeling was conducted in R version 3.6.0 using the package deSolve [Soetaert K, Petzoldt T, Setzer RW (2010). "Solving Differential Equations in R: Package deSolve." Journal of Statistical Software, 33(9), 1–25. doi: 10.18637/jss.v033.i09]. The R package was also used [Hamilton NE, Ferry M (2018). "ggtern: Ternary Diagrams Using ggplot2." Journal of Statistical Software, Code Snippets, 87(3), 1–17. doi: 10.18637/jss.v087.c03.] Code for the ecological model is available in the Zenodo repository at https://doi.org/10.5281/zenodo.4321797. Code for data analysis is available upon reasonable request.
Data analysis	Data analysis was conducted in R version 3.6.0.

For manuscripts utilizing custom algorithms or software that are central to the research but not yet described in published literature, software must be made available to editors and reviewers. We strongly encourage code deposition in a community repository (e.g. GitHub). See the Nature Research guidelines for submitting code & software for further information.

Data

Policy information about availability of data

All manuscripts must include a data availability statement. This statement should provide the following information, where applicable:

- Accession codes, unique identifiers, or web links for publicly available datasets
- A list of figures that have associated raw data
- A description of any restrictions on data availability

Source data are provided with this paper and are also available in the Zenodo repository at https://doi.org/10.5281/zenodo.4321797.

Field-specific reporting

Please select the one below that is the best fit for your research. If you are not sure, read the appropriate sections before making your selection.

☐ Life sciences ☐ Behavioural & social sciences ☒ Ecological, evolutionary & environmental sciences

For a reference copy of the document with all sections, see nature.com/documents/nr-reporting-summary-flat.pdf

Ecological, evolutionary & environmental sciences study design

All studies must disclose on these points even when the disclosure is negative.

Study description	This study involved laboratory experiments with bacterial populations and communities and modeling experiments done in R version 3.6.0. Lab experiments involved two strains of Escherichia coli and one strain of Salmonella enterica. Three lab experiments were performed: measuring growth rates in monoculture, growth rates in coculture, and measuring yields of each population to calculate changes in frequency in mutual invasibility experiments. The study design was not nested. For all lab experiments, three biological replicates were used for each population or community. For modeling experiments, no replicates were needed because the model was deterministic.
Research sample	We used two Escherichia coli K12 strains derived from the Keio collection [Baba, T. et al. Construction of Escherichia coli K-12 in-frame, single-gene knockout mutants: the Keio collection. Mol. Syst. Biol. 2, (2006)]. One E. coli strain is a methionine auxotroph, due to a metB deletion, and the other is an arginine auxotroph, due to an argA deletion. Both strains contain lacZ. We also used a strain of Salmonella enterica serovar Typhimurium LT2 that was evolved to secrete methionine and arginine (selection regime described in Methods).
Sampling strategy	We used three biological replicates for all experiments (measuring growth rates in monoculture, growth rates in coculture, and changes in frequency in mutual invasibility experiments). Growth rates were measured by reading the OD600 of the entire population. Frequencies were measured by diluting a subsample of the population (30µL) with serial dilutions and plating 100µL onto Petri dishes. We then counted between 30 and 300 colonies for each population. N = 3 was chosen because variability among replicates was low.
Data collection	Author SPH recorded growth rate data using a Tecan InfinitePro 200 plate reader, and frequency data by counting colonies on Petri dishes and recording the colony counts.
Timing and spatial scale	Communities were grown until all populations had reached carrying capacity. Experiments were conducted in 96-well plates with 200µL per well.
Data exclusions	No data were excluded from the analysis.
Reproducibility	All attempts to repeat the experiments were successful and are included in the manuscript. Several experiments were repeated on different weeks (labeled as different "batches" in the manuscript) and results were consistent across batches.
Randomization	Allocation of organisms into groups was random.
Blinding	Growth curves were analyzed computationally with a simple script to avoid any bias. Simulation results were deterministic and did not require blinding.

Did the study involve field work? ☐ Yes ☒ No

Reporting for specific materials, systems and methods

We require information from authors about some types of materials, experimental systems and methods used in many studies. Here, indicate whether each material, system or method listed is relevant to your study. If you are not sure if a list item applies to your research, read the appropriate section before selecting a response.

Materials & experimental systems

n/a	Involved in the study	
☒	☐	Antibodies
☒	☐	Eukaryotic cell lines
☒	☐	Palaeontology and archaeology
☒	☐	Animals and other organisms
☒	☐	Human research participants
☒	☐	Clinical data
☒	☐	Dual use research of concern

Methods

n/a	Involved in the study	
☒	☐	ChIP-seq
☒	☐	Flow cytometry
☒	☐	MRI-based neuroimaging
